# Potential Impact of Using Canadian Syncope Risk Score on Emergency Department Hospitalizations for Syncope

**DOI:** 10.5811/westjem.42019

**Published:** 2025-10-03

**Authors:** Andrea W. Harris, Lindsie LaBonte, Guido Massaccesi, Benoit Stryckman, Bennett A. Myers, Daniel B. Gingold, R. Gentry Wilkerson

**Affiliations:** * Johns Hopkins Hospital, Department of Emergency Medicine, Baltimore, Maryland; † University of Maryland School of Medicine, Baltimore, Maryland; ‡ University of Maryland School of Medicine, Department of Emergency Medicine, Baltimore, Maryland

## Abstract

**Introduction:**

Syncope is a common emergency department (ED) presentation and frequently results in low-yield hospitalizations. The Canadian Syncope Risk Score (CSRS) is a validated risk stratification score that identifies 30-day risk of serious adverse events for patients presenting with syncope. In this retrospective, cross-sectional study we aimed to evaluate syncope admissions with the CSRS to determine potentially unnecessary hospitalizations.

**Methods:**

We identified patient visits for syncope at 11 EDs from February 2019–January 2020. We excluded patients with additional serious diagnoses that would have independently required admission and those who were discharged. We then randomly sampled the remaining charts until finding 200 that met study inclusion criteria on full chart review. We retrospectively calculated CSRS via manual chart review and identified the proportion of patients with low-risk CSRS. We compared demographic characteristics between those with low- vs medium- and high-risk CSRS.

**Results:**

We identified 5,718 adult patients hospitalized for syncope. Of these patient visits 3,999 were initially excluded, 336 were sampled, and 200 included for analysis. Of these, 39% (77/200, 95% CI 32–46%]) were low risk (CSRS < 1). Patients with low-risk CSRSs were younger (61.2 years vs 70.6 years of age; absolute difference [AD] 9.4 years; 95% CI 4.8–13.9), less likely to have heart disease (1.3% vs 61.8%; AD 60.5%, 95% CI −69.4% to −51.5%), and more likely to have substance use disorder (14.3% vs 4.9%; AD 9.4%, 95% CI 0.7–18.1%).

**Conclusion:**

In this sample of patients hospitalized for syncope, 39% had low-risk Canadian Syncope Risk Score. Had the CSRS been used, these patients could have been safely discharged, as their estimated 30-day serious adverse event rate was < 1%. Wider adoption of the CSRS could potentially reduce unnecessary hospitalizations for patients with syncope.

## INTRODUCTION

### Background

Syncope, a brief, sudden loss of consciousness with spontaneous and complete return to baseline, is usually caused by transient global cerebral hypoperfusion.^1^ Syncope accounts for 1–3% of emergency department (ED) visits in the United States.^2^ Although its course is often benign, between 7–23% of patients will experience a serious adverse event within 30 days of hospitalization.^3^ However, 30–70% of ED visits for syncope result in hospitalization, likely because high-risk etiologies have historically been difficult to differentiate at ED presentation or they required investigations beyond the scope of the initial ED evaluation.^2^,^3^

The Canadian Syncope Risk Score (CSRS) is a scoring system developed by Thiruganasambandamoorthy et al^3^ in 2016 to risk-stratify patients presenting to the ED with syncope and predict the risk of serious adverse events (SAE) in the 30-day period following presentation. The CSRS does not explicitly indicate what an emergency physician should do with regard to disposition of a syncope patient but rather guides decision-making using 30-day risk of SAEs for a given range of scores. Scores range from −3 (lowest risk) to 11 (highest risk). Scores < 1 indicate low- or very low-risk of 30-day SAE (1.2% in initial development study^3^). The risk of SAEs in this study is similar to the risk profiles seen in other clinical decision tools commonly used in the ED, such as the HEART score (history, ECG, age, risk factors, and troponin) Wells score, and the Pulmonary Embolism Rule-out Criteria.^4^–^6^ A low-risk CSRS (< 1) would support a clinical decision of discharge from the ED with clear instructions for return in the event of worsening symptoms. Medium-, high-, and very high-risk syncope in the CSRS scoring system are associated with a > 1.2% risk of SAEs and death and, therefore, may require hospitalization for further monitoring and evaluation.^3^,^7^,^8^

The CSRS has been validated in a prospective, multicenter cohort study of nine Canadian hospitals,^7^ which found patients with low-(CSRS of −1 or 0) and very low-risk CSRS (< −1) had a SAE rate of 0.4% within 30 days of presentation. Another international validation of the CSRS in eight countries on three continents was completed in 2022, which found the 30-day SAE rate to be 0.6%.^8^ Further studies have confirmed the sensitivity and specificity of the CSRS in determining risk stratification in two Iranian EDs^9^ and one Australian ED.^10^

### Importance

The CSRS may have value in identifying low-risk cases of syncope that can be safely discharged from the ED, which would reduce the burden of avoidable hospitalization. To our knowledge, only one prior study, based in Australia, has examined the potential impact of CSRS use. This hypothetical modeling study found that 16.9% of hospitalizations could be avoided if clinicians began consistently using the CSRS instead of usual care.^12^ The potential impact of using the CSRS on a cohort of actual patients in the United States has not been previously evaluated.

### Goals of This Investigation

Our goal in this study was to identify the potential impact of using CSRS for patients hospitalized for syncope in whom a serious diagnosis was not identified during the initial ED evaluation. Using data from a large medical system in the US, we sought to determine the proportion of patients hospitalized for syncope who were determined to be low- or very low-risk for SAEs using the CSRS. The primary outcome was the proportion of hospitalized patients with a CSRS score < 1. These hospitalizations could have possibly been avoided had the CSRS been used during the ED evaluation.

**Population Health Research Capsule**

What do we already know about this issue?

*The Canadian Syncope Risk Score (CSRS) is a validated risk stratification score that identifies 30-day risk of serious adverse events for patients with syncope*.

What was the research question?

*We sought to determine the proportion of patients hospitalized for syncope who were low- or very low-risk using the CSRS*.

What was the major finding of the study?

*In our sample of patients hospitalized for syncope, 39% (77/200, 95% CI 32–46%) were low risk (CSRS < 1)*.

How does this improve population health?

*Wider adoption of the CSRS in the disposition decision could potentially reduce unnecessary hospitalizations for patients with syncope*.

## METHODS

### Study Design and Setting

This retrospective, cross-sectional, pilot observational study was performed at 11 EDs within the University of Maryland Medical System, including one academic and 10 community hospitals. The University of Maryland, Baltimore institutional review board (IRB) evaluated this study and determined it to be exempt from IRB review. We followed 10 of the 12 elements of medical record review studies as described by Worster et al, with the exception of blinding of the abstractors to the study objectives and formal testing of inter-rater reliability.^13^

### Selection of Participants

Using Epic (Epic Systems Corporation, Verona, WI), the electronic health record for the medical system, charts were reviewed for inclusion if patients 18 years or older presented to the ED with a diagnosis of syncope per *International Classification of Diseases, 10^th^ Rev*, codes (ICD-10 R55 for syncope found in the ED clinical impression as entered by emergency physicians at the time of evaluation, management, and disposition) between February 1, 2019–January 31, 2020. Data collection was completed from February–September 2023. We excluded patient visits if patients were discharged from the ED, if the CSRS could not be used due to its own exclusion criteria (ie, syncopal episode > 5 minutes in length, > 24 hours since syncopal episode, no loss of consciousness noted), if the patient was noted to have an independent serious diagnosis requiring admission, or if there was a mental status change or other condition that limited history-taking or documentation per the ED note.

We also excluded visits if ICD-10 codes from the ED clinical impression included a serious diagnosis requiring admission, including cardiac arrest, pulmonary embolism, myocardial infarction, respiratory failure, intracranial hemorrhage, and seizure. At this stage, visits were also excluded via ICD-10 code for mental status changes, intoxication, head trauma causing loss of consciousness, and pregnancy. Additionally, we excluded visits with ICD-10 codes of R55 but selected character strings indicating near syncope or pre-syncope as these diagnoses preclude use of the CSRS.

**Figure 1:**
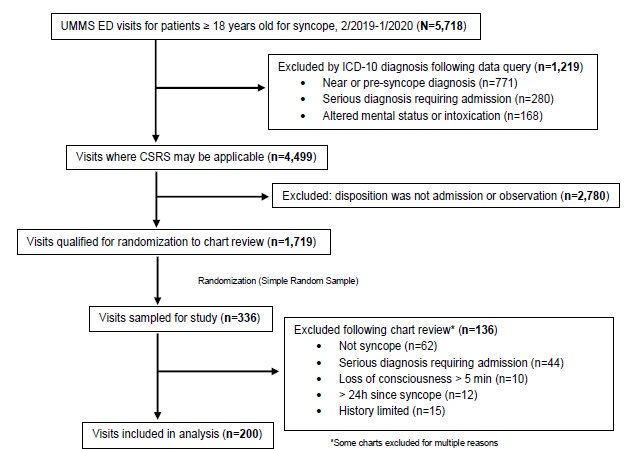
Flow diagram for patient visits with syncope. *UMMS*, University of Maryland Medical System; *ED*, emergency department; *ICD*, International Classification of Diseases; *CSRS*, Canadian Syncope Risk Score.

Following initial exclusions completed by query, visits were randomized (Figure 1) for selection for manual chart review. Cases were reviewed sequentially and excluded if additional exclusion criteria were discovered after review was completed. This process was completed until 200 charts were eligible to be included in the analysis. Because this was a retrospective pilot study primarily designed to explore patterns rather than test a specific hypothesis, we initially selected a convenience sample of 200 charts to allow for feasibility within our available timeframe. However, a post hoc power calculation was performed to assess the adequacy of this sample size. Assuming an acceptable baseline rate of 20% for low-risk CSRS scores among admitted patients with syncope, our sample size of 200 provides a 77.8% power to detect a 15% absolute difference (AD) from this proportion.

### Measurements

Data extraction was completed via chart review using a standardized abstraction instrument with Research Electronic Database Capture (REDCap) software (Vanderbilt University, Nashville, TN). Variables were precisely defined with an explicit protocol, and three medical student abstractors were trained on chart abstraction criteria. An initial pilot of 20 patient charts was performed and compared between extractors and attending emergency physician senior authors to ensure understanding of variable definitions; discrepancies during these pilot abstractions were resolved through consensus at regularly scheduled meetings to review coding rules. A senior medical student abstractor reviewed 5% of charts abstracted for quality review; just one data element was misclassified in that review and was corrected. Abstractors were not blinded to the study hypothesis.

### Outcomes

We calculated the CSRS values based on abstracted data from patient charts based on CSRS scoring criteria (Table 1), including systolic blood pressures (abnormal: < 90 or > 180 millimeters of mercury) elevated troponin (> 99^th^ percentile of normal population), abnormal QRS axis (< −30° or > 100°), abnormal QRS duration (> 130 milliseconds [ms]), and abnormal QTc (> 480 ms).^3^ All electrocardiograms (ECG) were interpreted by the treating clinician at the time they were obtained. If a patient had repeat vital signs, troponins, or ECGs measured during the time from ED arrival to ED disposition, any abnormal value could be used to accumulate CSRS points. If a troponin was not ordered or missing from a chart, it was presumed to be < 99th percentile for the patient population. This is consistent with the initial development of the CSRS, which calculated CSRS values by imputing normal troponins when they had not been ordered, and consistent with other previously published work on the topic.^3^,^7^,^8^

Syncope type (cardiac, vasovagal, or neither) was identified if the ED chart specifically identified either cardiac or vasovagal syncope as a diagnosis or if written portion of the chart included these specific terms; otherwise, syncope type was imputed as “neither.” History of structural or ischemic heart disease included coronary or valvular heart disease, cardiomyopathy, congestive heart failure, prior device implementation, or documented history of ventricular or atrial arrhythmias^3^ as identified by diagnoses in the patient’s prior medical history or as mentioned by the charting clinician in the history of present illness (HPI). Predisposition to vasovagal symptoms was identified either with a prior diagnosis of vasovagal syncope or mention in HPI of prior history of syncopal event triggered by a warm crowded place, prolonged standing, fear, emotion, or pain.^3^ If no documentation of heart disease history or vasovagal symptoms was documented, patients were assumed to have no such history.

Medical history components of interest (hypertension, diabetes, malignancy, seizure, stroke, substance use disorder, kidney disease on dialysis, previous syncopal episode) were identified by the treating clinicians and included if either mentioned in the ED chart HPI or diagnosis and medical history portion of the patient’s chart at time of ED visit. Associated symptoms of interest (diaphoresis, nausea, vomiting, abdominal pain, vision changes, shortness of breath, chest pain) were identified by the treating clinician and included if mentioned in the ED chart HPI. We identified patient sex using information in the electronic health record upon patient registration.

### Analysis

We calculated the proportion of patients with low- and very low-risk CSRS (total score < 1), and a 95% CI was obtained for all included visits, as well as for academic and community visits separately. We compared patient-level characteristics, including medical history and associated symptoms, between low- and medium-/high-risk CSRS groups as proportion of patients with each characteristic. We computed ADs of these proportions to compare these groups. We calculated CIs for all proportions with binomial exact calculations. Sensitivity analysis for troponin ordering status was completed by comparing the proportion of low-risk CSRS values for those with vs without troponin ordered. We performed data analysis using SAS v9.4. (SAS Institute Inc, Cary, NC) and Microsoft Excel v16.0 (Microsoft Corporation, Redmond, WA).

## RESULTS

### Characteristics of Study Subjects

A total of 5,718 adult patients presented to one of the study site EDs with a diagnosis of syncope (ICD-10 code R55) from February 1, 2019–January 31, 2020. After excluding patients with serious outcomes, seizure, mental status change, intoxication, head trauma, pregnancy, and presyncope, 1,719 had a disposition of admitted or placed in observation and qualified for randomization to chart review. Of these, 336 patient visits were randomly selected and reviewed. After further exclusions based on chart review, 200 charts were included in the analysis (Figure 1).

Mean patient age was 67 years, 101 (50.5%) were women, and 77 (35.5%) had a history of structural or ischemic heart disease (Table 2). Forty-one patients were hospitalized under inpatient status, and 159 were hospitalized under observation status. Electrocardiograms and at least one blood pressure reading were available for all patients included in the study. A troponin was not ordered for 16 patients: 10 low-risk CSRS visits and six medium- or high-risk CSRS visits.

### Main Results

**Figure 2:**
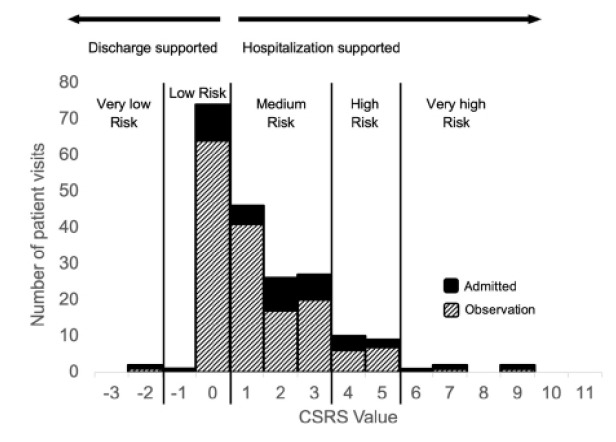
Histogram of Canadian Syncope Risk Score values for included patients. *CSRS*, Canadian Syncope Risk Score *mm Hg*, millimeters of mercury; *CSRS*, Canadian Syncope Risk Score; *ms*, millisecond.

In this sample of patients hospitalized for syncope, 1% (2/200) had a very low-risk CSRS and 38% (75/200) had a low-risk CSRS (Figure 2). In total, 39% (77/200) had calculated CSRS < 1, while 50% (99/200) had medium-risk CSRS (0 < CSRS < 4), 10% (19/200) had high-risk CSRS (3 < CSRS < 6), and 3% (5/200) had very high-risk CSRS (score > 5) (Figure 2).

For patients who presented to EDs at the academic hospital, 45% (15/33; 95% CI 28–64%) of those who were hospitalized for syncope had a CSRS < 1. For patients who were treated at community EDs, this value was 37% (62/167; 95% CI 30–45%). Among all hospitals, of the 77 patients who were hospitalized with low-risk CSRS, 11 were admitted to inpatient care and 66 were placed in observation status.

The most common components of the CSRS that were present and scored points were abnormal systolic blood pressure (42/200 (21%, 95% CI 16–27%), abnormal QTc interval (31/200 (16%), 95% CI 11–21%) (Table 3), and history of structural or ischemic heart disease (77/200 (39%), 95% CI 32–46%) (Table 4), Of 200 patients, 197 (99%) did not have a specific diagnosis of either vasovagal or cardiac syncope in the ED chart.

Hospitalized patients with very low- or low-risk CSRS were younger compared to patients with medium-, high-, or very high-risk CSRS (61.2 years vs 70.6 years; AD −9.4 years, 95% CI −4.8 to −3.9 years). Those with CSRS < 1 were less likely to have a history of either structural or ischemic heart disease (1.3% vs 61.8%; AD −60.5%, 95% CI −69.4% to −51.5%) and more likely to have a history of substance use disorder (14.3% vs 4.9%; AD 9.4%, 95% CI 0.7–18.1%) (Table 4).

**Figure 3:**
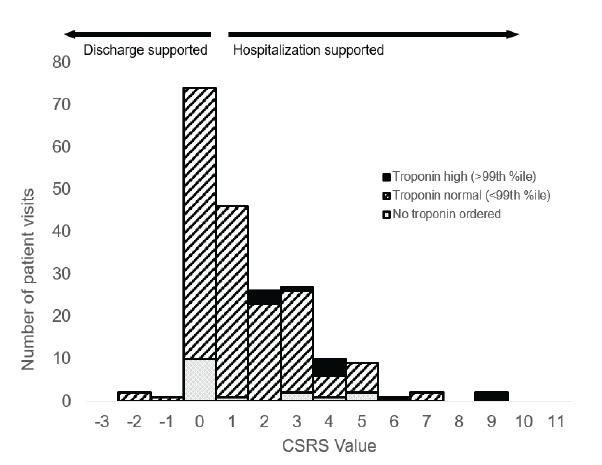
Histogram of Canadian Syncope Risk Score values by troponin status. *CSRS*, Canadian Syncope Risk Score

Of the included patients who did not have a troponin ordered, 63% (10/16, 95% CI 35–85%) were low-/very low-risk CSRS whereas 36% of those who had a troponin ordered were low-/very low-risk CSRS (67/184, 95% CI 29–44%); (AD 26%, 95% CI 1.4–51%). Of 184 patients with a troponin ordered, troponin levels were elevated in 11 (6.0%, 95% CI 2.6–9.4%) (Figure 3). Of these 11 patients, one died while hospitalized (CSRS 4) and one received a heart catheterization (CSRS 6). No patients with a low-risk CSRS had a positive troponin.

## DISCUSSION

In this retrospective analysis of ED patients hospitalized for syncope in a large, regional medical system, we found that 39% of patients hospitalized had a CSRS < 1, representing potentially dischargeable ED visits. Identification and discharge of these low-risk cases of syncope would help limit hospitalizations without increasing risk of serious adverse events. Although prior prospective studies have validated the CSRS and prospectively determined scores from patient visits,^3^–^5^ a unique contribution of this retrospective study is that it allows for observation of the impact that changing practice—using the CSRS on patients with a primary diagnosis of syncope rather than clinical judgment alone—may have on already stressed EDs and hospital systems. We believe this work should inform standardization of the approach to a syncopal patient using the CSRS.

These data indicate that emergency physicians may be more conservative in their decision-making than the CSRS suggests is necessary to avoid SAEs by hospitalizing patients presenting with syncope. Given the higher number of admitted syncope patients in the low-risk CSRS group, it appears that substance use, or factors related to it, may have contributed to an increased perceived risk of acute serious adverse events by the clinician. Concerns about the patient’s ability to follow up may have played a role, although other unmeasured clinical or complex, health-related social factors likely influenced these decisions as well.

While our primary analysis focused on the low-risk group, we acknowledge that some medium-risk patients may also be safely discharged in select cases, particularly in settings where shared decision-making, reliable outpatient follow-up, and ambulatory cardiac monitoring are available. However, this strategy is not yet standard practice, and there are currently no prospective data demonstrating the safety of routine discharge for medium-risk patients. Given this, we conservatively limited our analysis to the low-risk group. If some medium-risk patients could also be safely discharged, the potential benefits of CSRS implementation may be greater than our estimates suggest.

Given that syncope accounts for 1–3% of US ED visits,^2^ a potential decrease of 39% of hospitalizations for this population would be meaningful. Accordingly, a more informed decision-making process for ED evaluation of syncope has the potential to reduce costs substantially for patients and hospitals with a possible estimated costs savings of $627,599 from $1,132,908 to the hospital system using average direct variable costs.^14^,^15^ Further analysis of avoidable hospital costs and length of stay for patients with low-risk CSRS scores would be valuable.

One component of the CSRS that is unique among syncope risk stratification scores is the measure of clinician judgment of syncope etiology as being cardiac, vasovagal, or neither.^16^ This may be beneficial because most other externally validated risk scores have not been shown to perform better than clinician judgment alone.^17^ For nearly all visits in our study (197/200), the note writer did not specify which kind of syncope they suspected; for these diagnoses we imputed “neither” as the type of syncope. Our findings may indicate that emergency physicians are not regularly noting the etiology of syncope in their assessment once they have decided to hospitalize a patient based on other factors. Implementation of the CSRS may subsequently force clinicians to consider whether there is a likely etiology of syncope and be able to incorporate the CSRS into their disposition planning.

Future studies on the impacts of CSRS use would benefit from incorporating actual hospital costs and length of stay from patient visits into the analysis, including discharged patients who may have been hospitalized had the CSRS been used. A previous study in Australia found that using the CSRS compared to standard care could be a cost-effective intervention.^12^ Cost-effectiveness analysis in the US has yet to be published. Incorporation of data from multiple hospital systems or geographic areas may elucidate the extent of practice variation and the impact CSRS use could have in specific regions. A prospective study to determine the impacts of CSRS use in EDs would be valuable, particularly to understand how clinicians are using the score during patient evaluation and treatment.

## LIMITATIONS

The study is subject to limitations inherent to retrospective analyses; clinician hospitalization and discharge decisions as well as use of CSRS may vary if these were to be studied prospectively. Also, there is potential for bias, including misclassification and selection bias, due to retrospective manual chart review and reliance on ICD-10 codes for identifying inclusion and exclusion diagnoses. Specifically, we relied on ICD-10 codes recorded in the ED clinical impressions, which are typically documented by emergency clinicians at the time of patient evaluation, management, and disposition. Only conditions known to the physician at the time of ED-to-hospital admission were included. However, we acknowledge some patients admitted with low-risk CSRS scores may have had additional conditions or reasons for admission not captured in ICD-10 codes.

We excluded many patients whose scores were not calculable based on the limited information available in the ED chart; had the score been used prospectively, treating clinicians may have had adequate information to calculate a CSRS. There may have been reasons treating clinicians decided to hospitalize patients that were not captured in the chart review, such as concerns about follow-up outpatient care due to health-related social needs. This study also had limited ability to capture the clinician-decided syncope category of cardiac, vasovagal, or neither; chart review only identified cases as cardiac or vasovagal if the note specifically used this language or the clinician used a diagnosis code with one of these terms.

The analysis included 16 patients for whom a troponin level was not obtained, 10 in the very low/low-risk group and six in the medium-/high-/very high-risk group. However, a troponin is not required to calculate a CSRS; per the team that developed the score if a troponin is not done, the score for that component is zero.^3^ In the initial study from which the CSRS was developed, 52% of patients did not have a troponin measurement.3 In our study this was 8% of patients.

Our analysis was focused on a one-year period from February 2019–January 2020, prior to the start of the COVID-19 pandemic in the US, which was chosen to limit the impact of changing ED visit patterns on our analysis.^11^ The CSRS had been developed by this time, but international validation of the CSRS has been published since 2019.^8^ We do not know whether clinicians in the aggregate have altered their practice since 2019 to incorporate the CSRS in their evaluation of syncopal patients. The percentage of hospitalized syncopal patients that may be dischargeable could be lower than 39% if clinicians are now using the CSRS more often to make hospitalization and discharge decisions.

Our study focused only on EDs in a single state and a single hospital system. Practice variation is likely in other geographic areas, and the results of our study are not necessarily generalizable across the US or internationally. While charts were sampled based on the proportion of syncope-related ED visits at each hospital, the academic hospital may be slightly over-represented in our dataset. This could potentially have skewed our data, particularly if clinical practices differ between academic and community settings; however, the inclusion of both academic and community hospitals improves the generalizability of our findings.

Because we did not include in our analysis patients who were discharged, we could not draw conclusions about the impact of using the CSRS on patients who may have otherwise been discharged (ie, those who may have been discharged with high-risk CSRS). It is possible that universal use of CSRS could increase hospitalizations in this cohort, which would reduce potential hospitalization avoidance and cost savings. We did not examine the frequency of serious adverse events in this analysis; the validity of the CSRS has been demonstrated multiple times across institutions and countries in the literature,^7^–^10^ and we relied on these published estimates rather than re-evaluating the frequency of these events.

## CONCLUSION

This study showed that a substantial proportion of patients hospitalized for syncope from the ED had low risk of 30-day serious adverse events as identified by the Canadian Syncope Risk Score. Wider adoption of the CSRS in emergency departments may limit unnecessary hospitalizations for patients with syncope.

## Figures and Tables

**Table 1:** Canadian Syncope Risk Score components.^3^

Variable	Score if present
Predisposition to vasovagal symptoms*	−1
Heart disease history+	+1
Any systolic blood pressure < 90 mm Hg or >180 mm Hg	+2
Elevated troponin (> 99th percentile)	+2
Abnormal QRS axis (< −30 or > 100 degrees)	+1
QRS duration > 130 ms	+1
QTc > 480 ms	+1
ED diagnosis	
Vasovagal syncope	−2
Cardiac syncope	+2
Neither	0

* Symptoms triggered by being in a warm crowded place, prolonged standing, fear, emotion, or pain.

+ Ischemic or structural heart disease, including coronary artery disease, atrial fibrillation or flutter, congestive heart failure, or valvular disease.

*mm Hg*, millimeters of mercury; *ED*, emergency department; *ms*, millisecond.

**Table 2:** Baseline characteristics of patients hospitalized for syncope included in study.

Variable	Total N = 200
Age, years (SD)	67 (17)
Sex	
Female (%)	101 (51%)
Hospital type	
Academic (%)	33 (17%)
Community (%)	167 (84%)
Medical history	
Structural or ischemic heart disease (%)	77 (36%)
Hypertension (%)	133 (67%)
Diabetes (%)	47 (24%)
Malignancy (%)	34 (17%)
Seizure (%)	5 (3%)
Stroke (%)	25 (13%)
Substance use disorder (%)	17 (9%)
Kidney disease on dialysis (%)	6 (3%)
Previous syncopal episode (%)	42 (21%)

*SD*, standard deviations.

**Table 3:** Canadian Syncope Risk Score components by risk group.

Variable	Low-risk CSRS (n = 77) N (%)	Medium- or high-risk CSRS (n = 123) N (%)
Predisposition to vasovagal symptoms	2 (2.6%)	1 (0.8%)
Systolic blood pressure < 90 or > 180 mm Hg	0 (0%)	42 (34.2%)
Troponin > 99th percentile	0 (0%)	11 (8.9%)
Abnormal QRS axis (< −30 or > 100 degrees)	0 (0%)	34 (27.6%)
QRS duration >130 ms	0 (0%)	23 (18.7%)
QTc >480 ms	0 (0%)	31 (25.2%)
Emergency department diagnosis		
Vasovagal syncope	2 (2.6%)	0 (0%)
Cardiac syncope	0 (0%)	1 (0.8%)
Neither	75 (97.5%)	122 (99.2%)

**Table 4:** Comparison of patient-level characteristics between low-risk and medium- or high-risk Canadian Syncome Risk Score in sample.

Variable	Low-risk CSRS (n=177) N (%)	Medium- or high-risk CSRS (n=123) N (%)	Absolute Difference (95% CI)
Demographics			
Age, years (SD)	61.2 (18)	70.6 (14)	−9.4 (−4.8 to −13.9)
Sex: Female	39 (50.6%)	62 (50.4%)	0.2% (−13.2% to 13.7%)
Hospital type			
Academic	15 (19.5%)	18 (14.6%)	4.8% (−5.9% to 15.7%)
Community	62 (80.5%)	105 (85.4%)	−4.8% (−15.7 to 5.9%)
Medical history			
Structural or ischemic heart disease	1 (1.3%)	76 (61.8%)	−60.5% (−69.4% to −51.5%)
Hypertension	44 (57.1%)	89 (72.4%)	−15.2% (−28.8% to −1.6%)
Diabetes	13 (16.9%)	34 (27.6%)	−10.8% (−22.3% to 0.8%)
Malignancy	7 (9.1%)	27 (22.0%)	−12.9% (−22.6% to −3.1%)
Seizure	3 (3.9%)	2 (1.6%)	2.3% (−2.6% to 7.1%)
Stroke	6 (7.79%)	19 (15.45%)	−7.7 (−16.4% to 1.1%)
Substance use disorder	11 (14.29%)	6 (4.88%)	9.4% (0.7% to 18.1%)
Kidney disease on dialysis	1 (1.30%)	5 (4.07%)	−2.8% (−7.1% to 1.5%)
Previous syncopal episode	20 (26.0%)	22 (17.89%)	8.1% (−3.8% to 20.0%)
Symptoms on presentation			
Diaphoresis	15 (19.48%)	19 (15.45%)	4.0% (−6.9% to 14.9%)
Nausea	15 (19.48%)	24 (19.51%)	0.0% (−11.3% to 11.3%)
Vomiting	9 (11.69%)	15 (12.20%)	−0.5% (−9.7% to 8.7%)
Abdominal pain	8 (10.39%)	7 (5.69%)	4.7% (−3.3% to 12.7%)
Vision changes	3 (3.90%)	7 (5.69%)	−1.8% (−7.7% to 4.2%)
Shortness of breath	6 (7.79%)	14 (11.38%)	−3.6% (−11.8% to 4.6%)
Chest pain	11 (14.29%)	12 (9.76%)	4.5% (−4.9% to 13.9%)

*CSRS*, Canadian Syncope Risk Score; *SD*, standard deviations; *CI, confidence intervals*
